# Prevalence of marijuana use in pregnant women with concurrent opioid use disorder or alcohol use in pregnancy

**DOI:** 10.1186/s13722-021-00285-z

**Published:** 2022-01-06

**Authors:** Kimberly Page, Cristina Murray-Krezan, Lawrence Leeman, Mary Carmody, Julia M. Stephen, Ludmila N. Bakhireva

**Affiliations:** 1 Division of Epidemiology, Biostatistics and Preventive Medicine, Department of Internal Medicine, School of Medicine, University of New Mexico MSC 10 5550, Albuquerque, NM 87131 USA; 2 Department of Family and Community Medicine, School of Medicine, University of New Mexico, Albuquerque, NM USA; 3 Department of Obstetrics and Gynecology, School of Medicine, University of New Mexico, Albuquerque, NM USA; 4 The Mind Research Network a Division of Lovelace Biomedical Research Institute, Albuquerque, NM USA; 5 Department of Pharmacy Practice and Administrative Sciences, Substance Use Research and Education (SURE) Center, College of Pharmacy, University of New Mexico, Albuquerque, NM USA

**Keywords:** Alcohol, Cannabis, Marijuana, Opioids, Pregnancy

## Abstract

**Background:**

A quarter of pregnant women use alcohol, 6.5/1000 deliveries are affected by opioid use disorder (OUD), and the prevalence of cannabis use in pregnant women is increasing. However, marijuana co-exposure in polysubstance-using women is not well described.

**Methods:**

The well-characterized ENRICH-1 cohort (n = 251), which focused on the effects of two primary exposures of interest—opioids and alcohol, was used to (1) estimate the prevalence/frequency of marijuana use in those with OUD and/or alcohol use, and (2) examined correlates of marijuana use. Participants were classified into an OUD group (n = 125), Alcohol group (n = 69), and concurrent OUD and Alcohol (OUD + Alcohol) group (n = 57). Self-report and biomarkers ascertained substance use. Multivariable logistic regression identified correlates of marijuana use.

**Results:**

The prevalence of any marijuana use in pregnancy was 43.2%, 52.6%, and 46.4% in the OUD, OUD + Alcohol, and Alcohol groups, respectively. Correspondingly, weekly or daily use was reported by 19.4%, 21.0%, and 24.6% of participants. In the OUD and OUD + Alcohol groups, the proportion of women using marijuana was significantly higher in those taking buprenorphine (45.8% and 58.3%, respectively) compared to women using methadone (37.5% and 42.9%, respectively). Mean maternal age was lower in women who used marijuana in all three groups compared to non-marijuana users. Independent correlates of marijuana use (controlling for group, race/ethnicity, education, and smoking) were maternal age (adjusted Odds Ratio (aOR) per 5-year increment 0.61; (95% CI 0.47, 0.79)), and polysubstance use (aOR 2.02; 95% CI 1.11, 3.67). There was a significant interaction between partnership status and group: among women who were not in a partnership, those in the OUD and OUD + Alcohol groups had lower odds of marijuana use relative to the Alcohol group. For women in the Alcohol group, partnered women had lower odds of marijuana use than un-partnered women (aOR 0.12; 95% CI: 0.02, 0.68).

**Conclusions:**

Results indicate a relatively high prevalence and frequency of marijuana use in pregnant women being treated for OUD and/or women consuming alcohol while pregnant. These results highlight the need for ongoing risk reduction strategies addressing marijuana use for pregnant women receiving OUD treatment and those with alcohol exposure.

## Introduction

The opioid epidemic in the U.S. has affected people across the lifespan, including pregnant women and newborn children. In 2014, as many as 22.8% of Medicaid-enrolled pregnant women used prescription opioids during pregnancy [1]. Recent estimates demonstrated that more than 5% of pregnant women reported non-medical use of opioids in the past year [2]. The prevalence of OUD in pregnant women increased 333% between 1999 and 2014 to 6.5/1000 deliveries [3]. In some states (i.e., West Virginia, Vermont), the prevalence of OUD is more than 30/1,000 hospital deliveries, with New Mexico also experiencing high rates at 14.8/1000 [3, 4].

Numerous studies have demonstrated associations between prenatal opioid exposure and intrauterine growth restriction (IUGR), preterm delivery, and stillbirth [5]. It is unclear if prenatal opioid exposure, medications for the treatment of OUD (MOUD), neonatal opioid withdrawal syndrome (NOWS), and/or treatment for NOWS are associated with poor long-term neurodevelopmental outcomes or if adverse outcomes are primarily driven by adverse pre- and postnatal environmental factors. The MOTHER trial [6] and our earlier results from the ENRICH cohort [7] were mostly reassuring, showing few or no effects on early childhood neurodevelopmental outcomes. A recent study demonstrated that infants with NOWS are at increased risk of unplanned healthcare utilization during the first year of life [8] A key challenge in assessing obstetric and neonatal outcomes is the need to fully account for the effects of polydrug use, i.e., co-exposure with marijuana, alcohol, stimulants, or other substances.

Among the most common co-exposures, prenatal alcohol and tobacco are associated with adverse neurodevelopmental outcomes for the offspring [9, 10]. We demonstrated earlier that almost 25% of pregnant women with OUD reported binge drinking, and 85.8% reported tobacco use [10]. There is also emerging evidence showing prenatal marijuana exposure may be associated with adverse perinatal outcomes, including the risk of preterm birth, growth restriction, increased neonatal intensive care unit admission, and potential adverse neurodevelopmental outcomes [11–14]. While a retrospective cohort study of 191 maternal-infant dyads exposed to buprenorphine during pregnancy did not find any significant associations between the third-trimester marijuana use and adverse perinatal outcomes, marijuana co-exposure was associated with more severe NOWS [15].

The widespread use of medical marijuana and increasing legalization of recreational marijuana have been accompanied by increased reporting of its use among women of reproductive age [16, 17]. The National Survey on Drug Use and Health (NSDUH) demonstrated a significant increase in marijuana use in reported previous month use in both pregnant (from 3.4% to 7.0%) and non-pregnant women of reproductive age (from 6.8% to 11.2%) between 2002 and 2017 [18]. The most notable increase in marijuana use was in the first trimester of pregnancy (from 5.7% to 12.1%). Similarly, a cross-sectional study of pregnant women receiving prenatal care at Kaiser Permanente Northern California demonstrated the prevalence of any marijuana use increased from 2.0% to 3.4% between 2009 and 2017 [19]. Both studies also showed that the frequency of use substantially increased over time. In addition, in states with legalized medical marijuana, the rate of admissions for marijuana use in substance use treatment programs increased by more than fourfold in pregnant women [20]. However, the prevalence of marijuana use in pregnant women who receive care for opioid use disorder (OUD) or other substances, including alcohol, is not well characterized.

We conducted a secondary analysis of data obtained from pregnant women participating in a well-characterized cohort with primary exposure to MOUD and alcohol [21] to: (1) estimate the prevalence and frequency of marijuana use (by self-report and urine drug testing) in those with OUD and/or alcohol exposure and (2) examine correlates of marijuana in this cohort. Based on the existing literature, we hypothesized that (a) the prevalence of marijuana use will be higher in pregnant women who use opioids and/or alcohol in pregnancy compared to the national estimate from the general obstetric population; (b) prevalence estimates of marijuana use will be comparable from self-report and urinalysis; (c) prevalence will be higher in younger women.

## Methods

### Study design and population

The data were obtained from the ENRICH-1 cohort at the University of New Mexico (UNM), described elsewhere [21]. In brief, the ENRICH-1 study evaluated the effects of prenatal substance use through four prospective visits: (1) a baseline prenatal visit; (2) hospitalization for labor and delivery; (3) 6 months postpartum, and (4) 20 months postpartum. Participants were recruited from UNM general prenatal care clinics and the Milagro clinic, which specializes in prenatal care for women with substance use disorders (SUD). ENRICH-1 eligibility criteria included: (1) the mother was ≥ 18 years old; (2) a singleton pregnancy confirmed by ultrasound; (3) gestational age 12–35 weeks at enrollment; (4) intention to deliver at the UNM Hospital and remain in the Albuquerque metropolitan area for at least two years; and (5) the ability to provide written consent in English. Exclusion criteria were: (1) a fetal diagnosis of a major structural anomaly; (2) greater than occasional (> 1 positive urine drug screen (UDS) or self-report of greater than monthly) use of cocaine, crack cocaine, methamphetamines, or MDMA during the first trimester and any use of these substances during the second or third trimesters. The study was approved by the UNM Human Research Review Committee; all participants provided signed informed consent.

Based on data collected at the baseline visit, the participants were categorized into three groups based on the primary exposure of interest: the OUD group (n = 125), Alcohol group (n = 69), and concurrent OUD and Alcohol (OUD + Alcohol) group (n = 57). Unexposed controls enrolled in the ENRICH-1 cohort were excluded from this analysis since the prevalence of marijuana use could not be estimated in that group (which excluded any substance use). It is important to note that women enrolled in the ENRICH-1 cohort classified into the Alcohol group were not defined by the diagnosis of alcohol use disorder; they were identified from a general obstetrics population if they (a), scored ≥ 2 on the AUDIT-C screening questionnaire, and consumed at least three drinks per week on average in the time between their last menstrual period (LMP) and pregnancy recognition, or (b) had ≥ 2 binge episodes (≥ 4 drinks/occasion) during the same interval.

### Characterization of prenatal substance use

Alcohol use during pregnancy was assessed through both self-report and maternal ethanol biomarkers. Self-reported drinking was captured at the baseline visit through two Timeline Followback Calendars (TLFB): (1) 30 days centered on LMP (15 days before and after); (2) a 30-day period prior to the baseline visit. Quantity and frequency of reported alcohol on each day were converted into ounces of absolute alcohol per day (AA/day) [22]. In addition, five ethanol biomarkers were tested in maternal blood and urine collected at the baseline visit, including gamma-glutamyl transpeptidase, carbohydrate-deficient transferrin, phosphatidylethanol, urine ethyl glucuronide, and ethyl sulfate.

Substance use, including marijuana, was ascertained through both self-report and urine cannabinoid biomarkers (carboxy-THC). A structured questionnaire, based on the 2011 National Survey on Drug Use and Health [23], was administered to assess self-reported substance use between LMP and enrollment (on average, 23.0 gestational weeks). Street names of substances were provided to facilitate recall, and frequency of use was captured. Polysubstance use was coded as positive if women reported using at least one other substance, including stimulants, benzodiazepines, sedatives, prescription medications (Soma/Carisoprodol), or hallucinogens. In addition, a urine drug screen (UDS)-7 panel (amphetamines, barbiturates, benzodiazepines, cocaine, opiates, phencyclidine (PCP), cannabinoids/tetrahydrocannabinols (THC), and a nicotine metabolites panel (nicotine, cotinine, 3-hydroxycotinine, nornicotine, anabasine) were collected and analyzed at the U.S. Drug Testing Laboratory (Des Plaines, IL). Marijuana use was defined as *any* self-reported use between LMP and study enrollment or a positive result on UDS at the baseline visit. If participants were missing either self-report or UDS results for marijuana, prevalence estimates were based on the non-missing value. No participants were missing both values.

### Statistical analyses

Participant characteristics and co-exposures were summarized with descriptive statistics. Overall, marijuana use and non-use groups were compared with independent two-sample tests of means using a *t* test or Wilcoxon rank-sum test for normally and non-normally distributed data, respectively. Differences in categorical variables across the two groups were assessed with *χ*^2^ or Fisher’s exact tests, as appropriate. To compare marijuana use among the three exposure groups, independent logistic regression models including group, characteristic, and an interaction between the two were fit to the data. The interaction terms from these models were assessed for differential marijuana use across the groups by demographic characteristics. A multivariable logistic regression model was fitted to marijuana use which included group as an independent variable and other covariates found to be associated with marijuana use in bivariate analyses, those hypothesized to be associated a priori (i.e., using other substances), and known or potential confounders (i.e., age, ethnicity, race, educational status, partnership status, and tobacco use during pregnancy). Interaction terms were added to the model based on findings of differential effects of marijuana use across the groups in the analyses of characteristics and co-exposures previously described (i.e., group × marital status). Adjusted odds ratios (aOR) and corresponding 95% confidence intervals (95% CI) from the model were reported. All statistical analyses were performed in SAS 9.4 (SAS Institute, Cary, NC, 2012).

## Results

**Fig. 1:**
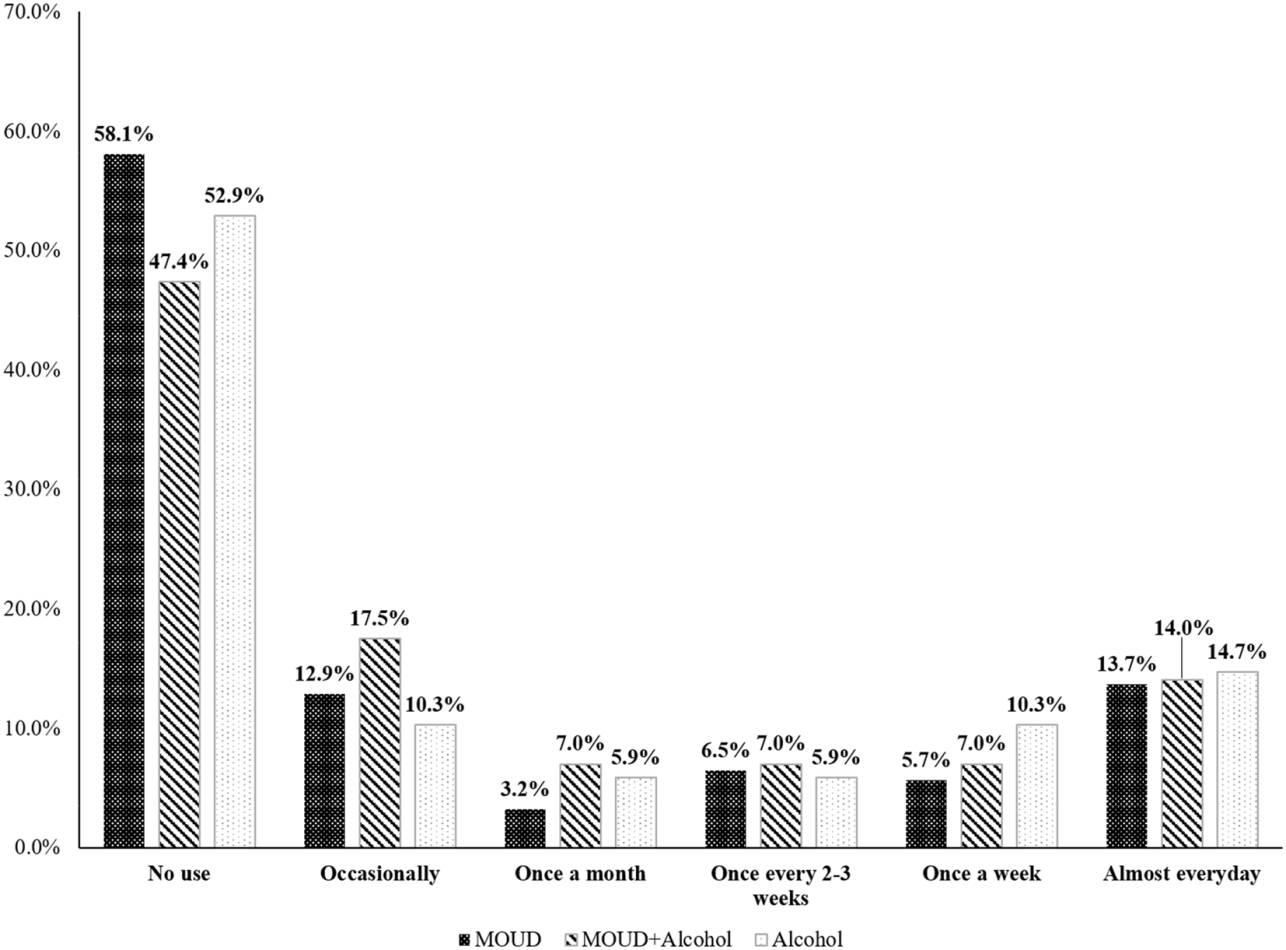
Frequency of marijuana stratified by study group

The sample included a high proportion of ethnic/racial minorities with 68.5% Hispanic/Latina and 6.4% American Indian women (Table 1). The majority (83.3%) of participants were enrolled in Medicaid. The overall prevalence of any marijuana use was 46.2% (45.8% by self-report and 24.0% by UDS). There were no differences at enrollment between marijuana users and non-users with respect to gestational age, partnership status, Hispanic ethnicity, race, attained educational level, employment status, or medical insurance type. Marijuana users were significantly more likely than non-users to be younger, to report the use of other substances, and to report higher alcohol use in early pregnancy (Table 1). The prevalence of stimulants (26.7% vs. 17.8%), benzodiazepines (23.3% vs. 14.8%), sedatives (7.8% vs. 3.0%), and tobacco (76.7% vs. 65.2%) was higher in marijuana users compared to non-users. No statistical differences between marijuana users and non-users were seen in self-reported heroin use (33.6% vs. 27.4%), self-reported opioid analgesics (27.6% vs. 20.7%), nor for opiates detected only by UDS (2.6% vs. 0.7%%). The frequency of marijuana use is shown in Fig. 1. Among 135 women who denied using marijuana, 2 (1.5%) had positive urine screen results, and of 114 women who reported marijuana use, 51 (44.7%) had negative urine screen results. There were two participants with missing self-reports and nine participants with missing urine results, and these were presumed to be negative. Overall, women in the OUD group had the lowest prevalence of any marijuana use (41.9% vs. 52.6% in the OUD + Alcohol group and 47.1% in the Alcohol group). The use of marijuana at least weekly was most prevalent in the Alcohol group (25.0%) in contrast to the two groups of women with OUD.

**Table 1:** Sociodemographic characteristics and substance use in the overall sample and by marijuana exposure during pregnancy (n = 251)

Characteristics	Overall	Marijuana exposure		
		Use (n = 116)	Non-use (n = 135)	p-value
	Mean (SD)	Mean (SD)	Mean (SD)	
Maternal age at enrollment (years)	28.4 (5.6)	27.0 (5.5)	29.5 (5.5)	< 0.001^1^
Gestational age at enrollment (weeks)	23.0 (7.1)	22.4 (7.3)	23.5 (6.9)	0.230^1^

	n (%)	n (%)	n (%)	p-value
Partnership status^a^				0.132^2^
Single/separated/divorced/widowed	108 (43.0)	56 (48.3)	52 (38.5)	
Married/cohabitating	142 (56.6)	60 (51.7)	82 (60.7)	
Ethnicity: Hispanic/Latina	172 (68.5)	82 (70.7)	90 (66.7)	0.494
Race				0.907^3^
African American	4 (1.6)	1 (0.9)	3 (2.2)	
American Indian/Alaska Native	16 (6.4)	7 (6.0)	9 (6.7)	
Multiracial/Other	17 (6.8)	8 (6.9)	9 (6.7)	
White	214 (85.3)	100 (86.2)	114 (84.4)	
Education level				0.570^2^
Less than high school	62 (24.7)	29 (25.0)	33 (24.4)	
High school graduate or GED	77 (30.7)	39 (33.6)	38 (28.1)	
Some college or higher	112 (44.6)	48 (41.4)	64 (47.4)	
Employed (at enrollment)^b^	93 (37.1)	38 (32.8)	55 (40.7)	0.210^2^
Health insurance status				0.684^2^
Medicaid	209 (83.3)	99 (85.3)	110 (81.5)	
Employer-based insurance	26 (10.4)	11 (9.5)	15 (11.1)	
Other^c^	16 (6.4)	6 (5.2)	10 (7.4)	
Type of MOUD				0.486^3^
Methadone	69 (27.5)	27 (23.3)	42 (31.1)	
Buprenorphine	108 (43.0)	54 (46.6)	54 (40.0)	
Methadone and buprenorphine	5 (2.0)	3 (2.6)	2 (1.5)	
Other opioid use^d^				
Heroin	76 (30.3)	39 (33.6)	37 (27.4)	0.303^2^
Opioid analgesics	60 (23.9)	32 (27.6)	28 (20.7)	0.217^2^
Opiates detected by UDS only	4 (1.6)	3 (2.6)	1 (0.7)	0.338 ^3^
Polysubstance use^e^	91 (36.3)	52 (44.8)	39 (28.9)	0.009^2^
Tobacco use	177 (70.5)	89 (76.7)	88 (65.2)	0.046^2^

	Mean (SD)	Mean (SD)	Mean (SD)	p-value
Average ounces of alcohol per day, LMP to Visit 1^f^	0.4 (1.2)	0.6 (1.5)	0.3 (1.0)	0.027^4^
AUDIT score	7.3 (9.0)	7.8 (8.6)	6.8 (9.4)	0.071^4^

*GED* General Educational Development, *MOUD* medication for opioid use disorder, *UDS* urine drug screen, *LMP* last menstrual period

^a^One partnership status is missing in the marijuana non-use group

^b^One employment status is missing the marijuana use group

^c^“Other” includes lack of insurance or self-purchased

^d^Categories are not mutually exclusive

^e^Polysubstance use defined as using at least one other substance including: stimulants, benzodiazepines, sedatives, prescription medications (Soma/Carisoprodol), or hallucinogens

^f^Average absolute ounces of alcohol per day across two 30-day timeline follow-back (TLFB) calendars

^1^P-value obtained from the two-sample *t* test

^2^P-value obtained from the chi-square test

^3^P-value obtained from the Fisher’s exact test

^4^P-value obtained from the two-sample Wilcoxon rank sum test

**Fig. 2:**
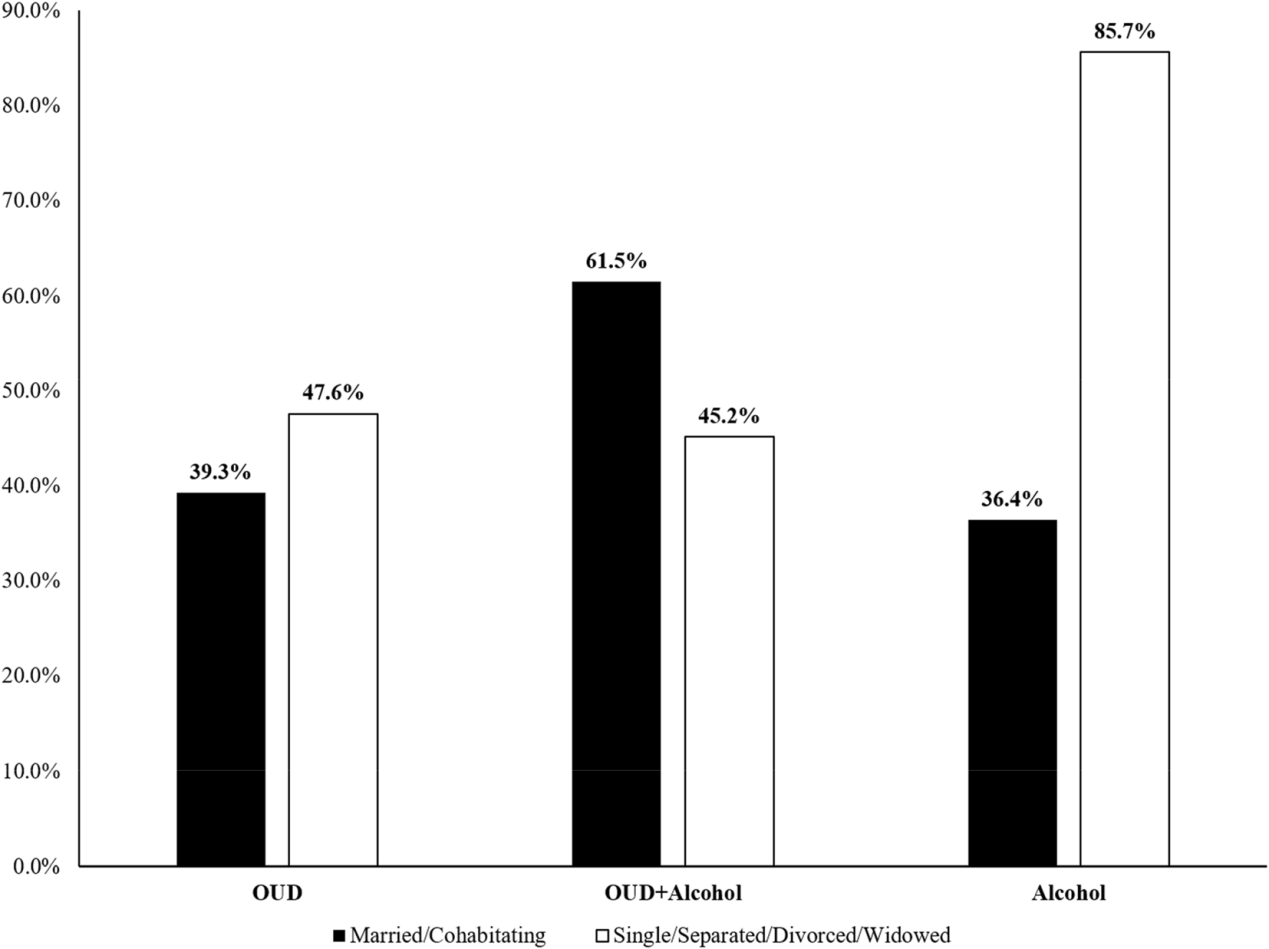
Self-reported marijuana use by marital status and group classification

Comparisons of the prevalence of marijuana use by the sociodemographic characteristics in the three study groups are shown in Table 2. No differences were found in marijuana use between groups by sociodemographic characteristics with the exception of partnership status. A significant interaction between study group and partnership status was found with respect to marijuana use: the highest proportion of marijuana users was in the Alcohol group, with 85.7% of un-partnered women reporting marijuana use compared to 36.4% of partnered women. In the OUD + Alcohol group, the reverse pattern was observed. Namely, marijuana use was higher in the partnered women (61.5%) compared to un-partnered women (45.2%). (Fig. 2).

**Table 2:** Prevalence of marijuana use during pregnancy by sociodemographic characteristics stratified by study group use (N = 251)

Characteristics	OUD (n = 125)		OUD + alcohol (n = 57)		Alcohol (n = 69)	
	Marijuana use (n = 54)	No marijuana use (n = 71)	Marijuana use (n = 30)	No Marijuana use (n = 27)	Marijuana use (n = 32)	No Marijuana use (n = 37)
	Mean (SD)	Mean (SD)	Mean (SD)	Mean (SD)	Mean (SD)	Mean (SD)
Maternal age at enrollment (years)	27.0 (5.9)	29.2 (5.3)	25.8 (4.3)	29.0 (5.2)	28.2 (5.6)	30.5 (6.2)
Gestational age at enrollment (weeks)	20.7 (6.6)	22.6 (6.6)	23.9 (7.7)	22.5 (7.3)	23.8 (7.7)	25.9 (6.9)

	n (%)	n (%)	n (%)	n (%)	n (%)	n (%)
Partnership status^1,a^						
Single/separated/divorced/widowed	30 (47.6)	33 (52.4)	14 (45.2)	17 (54.8)	12 (85.7)	2 (14.3)
Married/cohabitating	24 (39.3)	37 (60.7)	16 (61.5)	10 (38.5)	20 (36.4)	35 (63.6)
Ethnicity: Hispanic/Latina	42 (43.8)	54 (56.3)	24 (61.5)	15 (38.5)	16 (43.2)	21 (56.8)
Race						
African American	1 (100.0)	0 (0.0)	0 (0.0)	2 (100.0)	0 (0.0)	1 (100.0)
American Indian/Alaska Native	2 (28.6)	5 (71.4)	1 (50.0)	1 (50.0)	4 (57.1)	3 (42.9)
Multiracial/Other	3 (42.9)	4 (57.1)	1 (50.0)	1 (50.0)	4 (50.0)	4 (50.0)
White	48 (43.6)	62 (56.4)	28 (54.9)	23 (45.1)	24 (45.3)	29 (54.7)
Education level						
Less than high school	23 (46.9)	26 (53.1)	10 (55.6)	8 (44.4)	6 (60.0)	4 (40.0)
High school graduate or GED	12 (37.5)	20 (62.5)	12 (63.2)	7 (36.8)	5 (45.5)	6 (54.6)
Some college or higher	19 (43.2)	25 (56.8)	8 (40.0)	12 (60.0)	21 (43.8)	27 (56.3)
Employed (at enrollment)^b^	13 (33.3)	26 (66.7)	6 (66.7)	3 (33.3)	19 (42.2)	26 (57.8)
Health insurance status						
Medicaid	50 (42.4)	68 (57.6)	29 (54.7)	24 (45.3)	20 (52.6)	18 (47.4)
Employer-based insurance	3 (60.0)	2 (40.0)	1 (50.0)	1 (50.0)	7 (36.8)	12 (63.2)
Other^c^	1 (50.0)	1 (50.0)	0 (0.0)	2 (100.0)	5 (41.7)	7 (58.3)

*OUD* opioid use disorder, *GED* General Educational Development

^1^*p* < 0.01 (Wald test from logistic regression model for the interaction between marital status and group classification)

^a^One subject in the OUD group did not report partnership status

^b^One subject in the Alcohol group did not report employment status

^c^“Other” includes lack of insurance or self-purchased

Table 3 shows prevalence of marijuana use by MOUD and other substance use in the three groups. All (100%) women in the OUD and OUD + Alcohol groups were using prescribed methadone and/or buprenorphine. In the OUD group, under half (45.8%) of women prescribed buprenorphine used marijuana, whereas over half (58.3%) of women prescribed buprenorphine in the OUD + Alcohol group used marijuana, but this difference was not significant. Women who reported polysubstance use were more likely to report marijuana in all three groups (55.6%, 58.6%, and 58.8% in the OUD, OUD + Alcohol, and Alcohol groups, respectively) relative to non-polydrug users. While a slightly higher proportion of women reporting heroin use in the OUD + Alcohol group used marijuana (56.0%) in contrast to women who used heroin in the OUD group (49.0%), the interaction was not significant. Marijuana use was similar in women who reported opioid analgesic use or for whom opiates were detected only by UDS in all groups.

**Table 3:** Prevalence of marijuana use during pregnancy by MOUD and other substance exposures stratified by study group (N = 251)

Co-exposures	OUD (n = 125)		OUD + alcohol (n = 57)		Alcohol (n = 69)	
	Marijuana use (n = 54)	No Marijuana use (n = 71)	Marijuana use (n = 30)	No Marijuana use (n = 27)	Marijuana use (n = 32)	No marijuana use (n = 37)
	n (%)	n (%)	n (%)	n (%)	n (%)	n (%)
Type of MOUD					–	–
Methadone	18 (37.5)	30 (62.5)	9 (42.9)	12 (57.1)	–	–
Buprenorphine	33 (45.8)	39 (54.2)	21 (58.3)	15 (41.7)	–	–
Methadone and buprenorphine	3 (60.0)	2 (40.0)	0 (0.0)	0 (0.0)	–	–
Other opioids^a^						
Heroin	25 (49.0)	26 (51.0)	14 (56.0)	11 (44.0)	–	–
Opioid analgesics^b^	16 (53.3)	14 (46.7)	11 (52.4)	10 (47.6)	5 (55.6)	4 (44.4)
Opiates detected by UDS only	1 (50.0)	1 (50.0)	0 (0.0)	2 (100.0)	–	–
Polysubstance use^c^	25 (55.6)	20 (44.4)	17 (58.6)	12 (41.4)	10 (58.8)	7 (41.2)
Tobacco use	48 (47.1)	54 (52.9)	27 (54.0)	23 (46.0)	14 (56.0)	11 (44.0)

	Mean (SD)	Mean (SD)	Mean (SD)	Mean (SD)	Mean (SD)	Mean (SD)
Average ounces of alcohol per day, LMP to Visit 1^d^	0.0 (0.0)	0 (0.0)	1.0 (1.6)	0.7 (1.1)	1.3 (2.2)	0.6 (1.5)
AUDIT score^1^	1.1 (1.7)	0.6 (1.0)	11.8 (8.4)	15.6 (10.9)	14.2 (8.1)	10.3 (9.0)

*OUD* opioid use disorder, *GED* General Educational Development, *MOUD* medication for opioid use disorder, *UDS* urine drug screen, *LMP* last menstrual period

^a^Categories are not mutually exclusive

^b^Opioid analgesic use in the Alcohol group was directed by a physician and did not constitute misuse

^c^Polysubstance use defined as using at least one other substance including: stimulants, benzodiazepines, sedatives, prescription medications (Soma/Carisoprodol), or hallucinogens

^d^Average absolute ounces of alcohol per day across two 30-day timeline follow-back (TLFB) calendars

^1^*p* < 0.01 (Wald test from logistic regression model for the interaction between the AUDIT score and group classification)

After controlling for study group, ethnicity, race, education level, and tobacco use during pregnancy, independent correlates of marijuana use in all groups included maternal age—where marijuana use declined by 5-year increments in age (aOR 0.61; 95% CI: 0.47, 0.79) and polysubstance use—marijuana use increased among women with this exposure during pregnancy (aOR 2.02 (95% CI: 1.11, 3.67) (Table 4). The interaction between partnership status and group remained significant (Wald test *p* = 0.024) in multivariable analyses (Table 5). Among women who were not in a partnership, those in the OUD and OUD + Alcohol groups had significantly lower odds (88.8% and 91.5%, respectively) of using marijuana relative to the Alcohol group. Among women in the Alcohol only group, partnered women had 87.6% lower odds of reporting marijuana use than un-partnered women (aOR 0.12; 95% CI: 0.02, 0.68).

**Table 4:** Correlates of marijuana use: results of multivariable logistic regression

	aOR	95% CI
Maternal age (5-year increments)	**0.61**	**0.47, 0.79**
Polysubstance use^1^	**2.02**	**1.11, 3.67**
Hispanic ethnicity vs. non-hispanic	1.24	0.65, 2.36
Race^2^		
American Indian/Alaska Native vs. White	1.14	0.32, 4.15
Other vs. White	0.92	0.33, 2.61
Education		
< HS vs. ≥ College	0.91	0.46, 1.80
HS graduate/GED vs. ≥ College	0.87	0.43, 1.79
Tobacco use	1.80	0. 88, 3.68

Bold values indicate a statistically significant association

**Table 5:** Interaction between partnership status and study group on marijuana use

	Partnership status			
	Single/separated/divorced/widowed		Married/partnered	
	aOR	95% CI	aOR	95% CI
OUD	**0.11**	**0.02, 0.60**	0.68	0.28, 1.68
OUD + alcohol	**0.09**	**0.02, 0.50**	1.45	0.49, 4.33
Alcohol	1.00		1.00	

Bold values indicate a statistically significant association

*aOR* adjusted odds ratio, *GED* General Educational Development, *OUD* opioid use disorder

^1^Polysubstance use defined as using at least one other substance including: stimulants, benzodiazepines, sedatives, prescription medications (Soma/Carisoprodol), or hallucinogens

^2^Due to small frequencies, race was recategorized as White, American Indian/Alaska Native, Other Race = Black/Multiracial/Other race

## Discussion

The results of this study indicate a very high proportion (> 40%) of pregnant women, who are in treatment for OUD with or without concurrent alcohol consumption, used marijuana early in pregnancy. These estimates are substantially higher than recent (2017) estimates of past month marijuana use in pregnant and non-pregnant women in the general population surveyed in the NSDUH (7.0% and 11.2%, respectively [18], and the 3.4% rate of any use during pregnancy in Northern California [19]). A study in Washington state examined the effect of marijuana legalization on alcohol and drug use in pregnant/parenting women. Subjects were women attending a three-year program serving those who use alcohol and/or drugs during pregnancy, and the study found women exiting the program in the post-legalization period were twice as likely to report using marijuana in the 30 days before exiting than women in the pre-legalization cohort [24]. Moreover, post-legalization, women were twice as likely to continue use from program enrollment to exit.

Increased marijuana use among pregnant women may be associated with low perceived risk [25, 26]. Women often have contradictory views: on the one hand, trying to decrease use when they become pregnant, but at the same time believing that marijuana is safe, ‘natural,’ and a way to mitigate nausea and vomiting during pregnancy [26–28]. Women report their information sources about marijuana use include non-medical and medical sources [29, 30]. Some women with OUD also believe marijuana may help manage opioid dependence and withdrawal [31]. The prevalence of marijuana use among pregnant women with OUD and some alcohol use in this study was twice that reported by people with a chronic medical condition surveyed in the 2016 and 2017 Behavioral Risk Factor Surveillance System (BRFSS): 21.9% of those aged 18–34 with any chronic condition reported marijuana use versus 13.1% of people surveyed with no medical condition [32]. In general, among people with and without medical conditions, marijuana use has become more acceptable for both recreational and medical reasons; 7.3% of adult respondents in a national online survey agreed that marijuana use is somewhat or completely safe during pregnancy [33].

In light of the paucity of studies demonstrating safety in pregnancy, particularly concerning is the substantial proportion of women (~ 14% in both groups) who used marijuana almost daily, as contrasted to ~ 31% of patients using once a week or less during pregnancy. It should be noted that women in the Alcohol group reported light-to-moderate/intermittent alcohol use during pregnancy (1.5 drinks/week, on average, 30 days before enrollment).

Interestingly, prevalence estimates were higher per maternal self-report than urinalysis. We attribute this difference to a shorter detection window on urinalysis vs. the reporting timeframe (use between the LMP and the baseline visit – on average 22.98 (SD = 7.09) weeks). Urinary elimination of THC might vary depending on the quantity and frequency of marijuana use, creatinine levels, body mass index, and individual differences in metabolism. Typically, a rapid decrease is seen during the first three days of abstinence; however, subjects with high exposure levels might have detectable THC concentrations for several weeks [34]. Self-reported measures have the advantage of being noninvasive and enabling evaluation over longer time periods in contrast to biomarker assessments. The validity of self-reported drug use can vary by setting, population, and interviewer traits [35]. Validity of self-reported marijuana use has been shown to be high (agreement 81.1% and sensitivity 91.2% [36]. And, in drug treatment settings, self-reported opioid use has been shown to be high (sensitivity 99%) [37]. We have previously shown that pregnant women in a substance use treatment program generally under-report substance use, but opioids and marijuana are more accurately reported [38]. Specifically, sensitivity of self-report was 100% for methadone, 83.3% for buprenorphine, 58.3% for other opioids, and 57.9% for marijuana as opposed to amphetamines (37.5%), benzodiazepines (40%), and cocaine (47.4%) [38].

Younger maternal age, using other substances, and partnership status were independently associated with marijuana use in our study, consistent with other studies [18, 19]. Un-partnered women with OUD, with and without alcohol use during pregnancy, demonstrated a lower prevalence of marijuana use compared to women who just used alcohol alone. We observed, among women who were partnered (married or cohabitating), those with a history of both OUD and alcohol use were more likely to use marijuana than women who used alcohol alone. Few studies have examined marijuana use among pregnant women who have used other substances; however, our findings differ from other studies of pregnant women, wherein married women were less likely to use marijuana [25, 39] or opioids [40] compared to unpartnered (unmarried, divorced, separated, or widowed) women. Differences in marijuana use by marital status may be related to reasons for use, such as recreational, medical, or ease of access. In a study examining alcohol use in cannabis users (with and without a medical recommendation) Subbaraman et al. [41] found no differences in cannabis use by marital status. Partnership factors are known to influence substance use patterns. Some studies have found newly married men and women tend to reduce substance use over time [42, 43]. However, women in relationships with drug-using men may be more likely to use and misuse substances [44, 45]. Our findings point to the need to examine social factors specific to marijuana use in pregnant women with a history of substance and/or alcohol use.

Women in our study demonstrated a high prevalence of polysubstance use (36.3%), which is consistent with other reports [25, 40], and marijuana use was over twice as likely for women who reported polysubstance use in comparison to women who did not report polysubstance use. Concurrent tobacco use was particularly high (70.5%) in all women, and both tobacco and estimated alcohol consumption were higher among those who concurrently used marijuana. A study of over 160,000 pregnant women admitted for substance use treatment demonstrated that alcohol and marijuana were the most common co-exposures [46]. Polysubstance use is of significant concern among pregnant women. We have previously shown that the risk of growth deficiencies (i.e., head circumference < 10^th^ percentile) was substantially increased in polydrug users compared to abstinent controls [47]. Additionally, polysubstance exposure was found to be the most significant predictor of NOWS severity in buprenorphine-exposed neonates [48].

The findings of this analysis should be interpreted in light of several limitations. First, while the sample size of this study was large (251 participants), results cannot be generalized to all pregnant women who use substances in pregnancy since the patients were recruited from a comprehensive multi-disciplinary prenatal clinic at UNM focused on SUD. Further, results cannot be generalized to patients with OUD who do not receive MOUD who may have even higher rates of marijuana use. Second, we could not examine secular trends in the prevalence of marijuana use over time. In New Mexico, medical marijuana use was legalized in 2007, while recreational use remained illegal but was decriminalized in 2019. (In early 2021, recreational use was legalized.) Thus, since most data were obtained before 2019, estimates are likely to be conservative. Third, we acknowledge a relatively small sample size in the OUD + Alcohol and Alcohol groups and limited generalizability to patients with different alcohol consumption patterns, including women with alcohol use disorder. Strengths of the study include a prospective cohort design and state-of-the-art characterization of prenatal substance use with repeated prospective interviews and comprehensive batteries of biomarkers for alcohol, tobacco, and illicit drug use.

Future studies should specifically examine the prevalence of marijuana use in pregnant women with chronic use of opioid analgesics and those with OUD who are not receiving methadone or buprenorphine. Opioid use disorder has been listed (since 2018 and not overlapping with enrollment in this study) as one of the 28 indications for medical marijuana in New Mexico, and pregnancy is not an exclusion criteria, which may result in pregnant woman with OUD continuing or initiating medical marijuana [49]. Given the unique impact of the opioid epidemic on rural/under-served areas, it would be important to characterize the pattern of substance use in rural vs. urban areas and examine trends and motivations over time and as a function of marijuana legalization laws. A new preclinical research finding indicates that simultaneous exposure to alcohol and cannabinoid compounds increases the risk of birth defects in a murine model, indicative of a compounding interaction [50]. This research also demonstrated common signaling pathways for such interactive effects on fetal mice development. Future studies of adverse perinatal and pediatric neurodevelopmental outcomes associated with intrauterine exposure to opioids and alcohol need to characterize marijuana co-exposure carefully, given the potential additive effects.

The American College of Obstetricians and Gynecologists (ACOG) recommends that pregnant women “reporting marijuana use should be counseled about concerns regarding potential adverse health consequences of continued use during pregnancy… [and] should be encouraged to discontinue marijuana use” [51]. While little is known about the direct effects of marijuana use on the developing brain (based on restricted access to marijuana for research studies), cannabinoid receptors are found throughout the brain, and the scant work that has been performed demonstrates broad alterations to brain function with cannabinoid exposure [52–55]. As legalization of marijuana becomes more widespread and the percentage of THC in marijuana products continues to increase, additional information about the effects of prenatal marijuana exposure will need to be obtained. Moreover, ACOG also recommends that alternative therapeutic approaches with better pregnancy-specific safety data be considered instead of medical marijuana use. The American Academy of Pediatrics (AAP) also advises against the use of marijuana or other cannabinoid-containing products either for recreational or medical reasons, including nausea and vomiting during pregnancy. AAP recommends universal screening, brief intervention, and referral for treatment [56]. Currently, the ACOG, AAP and the Surgeon General recommend women to abstain from marijuana use completely if they are trying to conceive, pregnant or lactating. There remain significant gaps in knowledge and research however. For instance, there is not substantial data to conclude that abstinence from marijuana use will decrease negative perinatal/pediatric outcomes as it does for cigarette [57] and alcohol exposure [58]. Nevertheless, a harm reduction approach would include consideration of decreasing use in the high proportion of women with near-daily use.

## Conclusions

Results of this study indicate a high prevalence of marijuana use in pregnant women receiving MOUD and non-abstainers of alcohol during pregnancy, and a high proportion of these women use marijuana regularly. Our findings are concerning due to the risks of polysubstance use to both mother and fetus. Further research and evaluation of programs to reduce marijuana use in pregnant women, especially those with OUD or who engage in alcohol consumption, are needed.

## Abbreviations

AAP: American Academy of Pediatrics
ACOG: American College of Obstetricians and Gynecologists
aOR: Adjusted odds ratio
BRFSS: Behavioral Risk Factor Surveillance System
CI: Confidence intervals
GGT: Gamma-glutamyl transpeptidase
LMP: Last menstrual period
MDMA: Methylenedioxymethamphetamine
MOUD: Medications for the treatment of opioid use disorder
MOUD: Medications for the treatment of OUD
NIH: National Institutes of Health
NOWS: Neonatal opioid withdrawal syndrome
NSDUH: National Survey on Drug Use and Health
OUD: Opioid use disorder
PCP: Phencyclidine or phenylcyclohexyl piperidine
SUD: Substance use disorders
THC: Tetrahydrocannabinol
TLFB: Timeline Follow-back Calendars
UDS: Urine drug screen
UNM: University of New Mexico

## Declarations

### Availability of data and materials

The datasets generated and/or analyzed during the current study are not publically available due to lack of data sharing acknowledgement of de-identified data in the consent and IRB protocol. The request for data sharing can be considered on a case-by-case b asis with a formal data sharing agreement between institutions.
